# Bridging AI innovation and healthcare: scalable clinical validation methods for voice biomarkers

**DOI:** 10.3389/fdgth.2025.1575753

**Published:** 2025-07-03

**Authors:** Agnieszka Ewa Krautz, Jörg Langner, Florian Helmhold, Julia Volkening, Alina Hoffmann, Claudio Hasler

**Affiliations:** ^1^ PeakProfiling GmbH, Berlin, Germany; ^2^ Institute of Medical Psychology and Behavioural Neurobiology, University of Tübingen, Tübingen, Germany

**Keywords:** voice biomarkers, artificial intelligence, clinical validation, healthcare implementation, mental health

## Abstract

The integration of artificial intelligence (AI) in voice biomarker analysis presents a transformative opportunity for objective and non-invasive diagnostics in healthcare. However, clinical adoption remains limited due to challenges such as data scarcity, model generalizability, and regulatory hurdles. This perspective article explores effective and scalable methods for clinical validation of voice biomarkers, emphasizing the importance of proprietary technology, high-quality, diverse datasets, strong clinical partnerships, and regulatory compliance. We propose a multifaceted approach leveraging proprietary AI technology (Musicology AI) to enhance voice analysis, large-scale data collection initiatives to improve model robustness, and medical device certification to ensure clinical applicability. Addressing technical, ethical, and regulatory challenges is crucial for establishing trust in AI-driven diagnostics. By combining technological innovation with rigorous clinical validation, this work aims to bridge the gap between research and real-world implementation, paving the way for AI-powered voice biomarkers to become a reliable tool in digital healthcare.

## Introduction

Clinical validation is a critical process in healthcare that ensures medical devices, diagnostic tests, or treatments (in short interventions) are both effective and safe when applied in real-world clinical settings. This process involves rigorous evaluation to confirm that the intervention performs as intended and delivers expected clinical outcomes, maintaining patient safety and treatment efficacy [U.S. Food and Drug Administration (FDA), 1993–2017] (1). Effective clinical validation methods are characterized by their ability to produce accurate and consistent results, comprehensively evaluate all relevant aspects—including safety, efficacy, usability, and potential risks—and adhere to regulatory standards set by agencies such as the FDA in the US or European Medicines Agency (EMA) within the European Union. Equally important are scalable methods, which enable the widespread adoption of healthcare innovations, ensuring resource efficiency, and leveraging advanced technologies such as automation and data analytics (1).

Voice, with its unique properties, when analysed with artificial intelligence (AI) has the potential to become a powerful biomarker for various health conditions. As a non-invasive and nonintrusive approach, it enables remote diagnosis and monitoring through any phone or digital device, meeting the criteria for safety, usability, and accessibility. Additionally, it is potentially independent of variables such as language, geography, and age, supporting its applicability across diverse populations. The AI-driven analysis of voice data ensures secure, encrypted patient identification and integrates seamlessly with established clinical protocols set out by the regulatory agencies (2, 3). Despite the numerous benefits of integrating voice biomarkers into clinical practice, their adoption in real-world settings remains limited (e.g., 4–8). In this perspective article, we first explore the historical and conceptual foundations of voice biomarkers, before moving to the challenges of integrating voice biomarkers into clinical practice, followed by a number of solutions to these challenges directly derived from our own research and practice.

## Historical and conceptual foundations of voice biomarkers

The use of voice as a diagnostic tool has a long history in medicine, dating back to Hippocrates, who noted that voice changes could indicate underlying health conditions. More recently, physicians observed that disorders such as Parkinson's disease, depression, and respiratory illnesses could alter speech patterns, including pitch, rhythm, and articulation. Before the advent of AI, clinical speech analysis relied on subjective auditory assessments by trained professionals and objective acoustic measurements using tools like spectrograms and frequency analyzers.

In the mid-20th century, advancements in phonetics and speech science led to more structured approaches in analyzing voice abnormalities. Researchers identified measurable vocal markers associated with various conditions, such as increased jitter and shimmer in Parkinson's disease and slowed speech rate in depression. However, traditional methods were often labor-intensive, requiring manual analysis and expert interpretation (9).

The introduction of AI and machine learning in the 21st century revolutionized the field by automating voice analysis and uncovering complex patterns beyond human perception. Early AI applications in voice diagnostics focused on neurodegenerative diseases and psychiatric disorders. The emergence of deep learning further enhanced the accuracy and scalability of voice biomarkers, allowing for real-time analysis across diverse populations.

Most recently, AI-driven voice biomarkers have expanded beyond neurological and psychiatric conditions to include cardiovascular diseases, respiratory illnesses, and even COVID-19 detection (10–12). However, despite significant progress, the clinical adoption of voice biomarkers remains limited due to concerns about model generalizability, regulatory approval, and clinician trust (e.g., 4–8).

## Challenges of integrating voice biomarkers into clinical practice

Two recent systematic reviews by Meehan et al. (7) and Salazar de Pablo et al. (8) provide comprehensive comparisons of diagnostic models (which identify the presence of a psychiatric condition^1^), prognostic models (which predict the future onset or course of a condition), and predictive models (which estimate the likely response to a treatment). Out of 89 models included in the review by Salazar de Pablo et al. (8), the researchers mention one that was considered for implementation. Similarly Meehan et al. (7), out of identified 308 models, demonstrated that one published diagnostic model had its potential utility in clinical practice formally assessed. This indicates a significant gap in the clinical adoption of AI models and voice biomarkers for mental health.

Several factors may contribute to the above drafted situation. Martin and Rouas (6) argue that the primary barrier to integrating voice biomarkers into clinical settings is the lack of trust among clinicians. Furthermore, according to the authors the heterogeneity of symptoms further complicates the use of AI in mental health diagnostics. For instance, Fried and Nesse (13) found that among 3,703 patients diagnosed with major depressive disorder (MDD), there were 1,030 distinct symptomatic profiles, many of which had minimal overlap. Such variability makes it difficult to develop AI models that generalize effectively across diverse clinical populations. To address this challenge, the authors propose a shift in research focus toward the automatic estimation of psychiatric symptoms rather than diagnosing conditions. This approach, they argue, better reflects the complexity of mental health, where disorders like depression rarely occur in isolation and often present with comorbidities that influence speech patterns.

Additional challenges are highlighted by Berisha and Liss (4). As a primary concern they mention the lack of large-scale data, which leads to models that perform well in controlled conditions but fail to generalize in broader clinical applications. They also emphasize the importance of developing theoretically grounded models rather than just relying on purely data-driven approaches, particularly in contexts where data scarcity and the heterogeneity of conditions like depression may undermine model accuracy. To address these issues, they propose an analytical pipeline that calls for a closer collaboration between clinical speech scientists and data scientists. They further elaborate that the focus should be on creating models that account for the specific contexts of use—ranging from diagnostics and non-specific risk assessment to longitudinal tracking and the development of digital therapeutics.

Also, Fagherazzi et al. (5) highlight several key challenges that must be addressed for the efficient use of voice technology in healthcare. On the technical side, they stress the importance of creating and sharing extensive databases of high-quality audio recordings which are linked with clinical data. They advocate for increased harmonization and standardization of audio data across studies, the development of universal vocal biomarkers that transcend language, accent, age, and culture differences. On the ethical side, they stress the importance of secure data collection and storage, reliance on gold-standard clinical data for training algorithms, and transparency in defining the types and frequency of data collected. They also highlight the need to protect personal data, as voice is considered non-anonymous under Article 4.1 of the General Data Protection Regulation of the European Union (GDPR EU). To advance voice technology from research to clinical practice, they propose various study types, including proof-of-concept studies, replication studies, qualitative studies with co-design sessions, usability and pilot studies, and clinical utility evaluations, such as randomized controlled trials and real-world evaluation studies.

Finally, according to a recent review by Alhuwaydi (14), AI models show diagnostic accuracy ranging from 21% to 100% for various conditions like schizophrenia and cognitive impairment. Also, AI-driven personalized treatment plans, virtual therapists and chatbots have demonstrated early success in therapy. However, limitations in the generalizability of AI models, often due to reliance on homogenous datasets, remain a challenge. Furthermore, the author argues that ethical concerns, including data privacy, algorithmic bias, and the lack of empathy in AI-driven care, limit broader adoption of voice biomarkers. These challenges are compounded by evolving legal frameworks regarding AI accountability in healthcare. To address some of these issues, Alhuwaydi (14) stipulates that future research should focus on long-term studies, larger sample sizes, and culturally sensitive AI models to optimize its potential in mental health care.

In summary, while voice biomarkers and AI models hold great promise for mental health diagnostics and treatment, their clinical adoption remains limited due to several interrelated challenges. Trust among clinicians, symptom heterogeneity, data scarcity, and the need for theoretically grounded models all contribute to the slow transition from research to practice. Additionally, ethical concerns, regulatory uncertainties, and the necessity for standardized, high-quality datasets further complicate implementation.

## Discussion—bridging AI innovation and healthcare implementation

Our research and practice corroborate many of the limitations identified by the authors of the studies mentioned above. To overcome these challenges we follow a multifaceted approach that integrates cutting-edge technology, large-scale data collection, strong clinical collaborations, and regulatory compliance. In the discussion, we explore these four key solutions: (1) leveraging proprietary technology and complex feature engineering based on knowledge from Musicology (i.e., Musicology AI) to enhance voice biomarker analysis; (2) addressing the urgent need for larger, more diverse and high quality medical voice datasets; (3) strengthening clinical partnerships to support large-scale studies; and (4) achieving medical device certification to ensure regulatory approval and real-world applicability.

In our view, a key factor in improving voice-based diagnostics is the reliance on proprietary technology and informed feature sets, which enable a more sophisticated approach to data analysis. Existing studies on voice biomarkers rely on a limited set of acoustic features (Table 1), often analyzed using open-source tools such as openSMILE (https://www.audeering.com/de/research/opensmile/), VOICEBOX (https://voicebox.metademolab.com/), or Praat (17). While these tools offer valuable insights, they appear to struggle capturing the full complexity of voice-based signals relevant to medical diagnosis. For instance, research on conditions like heart failure, multiple sclerosis (MS), and lung cancer has mainly focused on features such as jitter, shimmer, loudness, MFCC, and F0 variability. Jitter and shimmer, key acoustic features reflecting variations in frequency and amplitude, respectively, have significantly improved the diagnostic accuracy and predictive ability of AI-driven diagnostic models, particularly in neurological disorders. However, a limited set of features and traditional methods may miss deeper, musicologically informed patterns that enhance diagnostics.

**Table 1:** Examples of acoustic features investigated across various health conditions.

Condition	Heart failure/pulmonary hypertension	Multiple sclerosis	Lung cancer
Acoustic features	Jitter	Jitter	Jitter
	Shimmer	Shimmer	Shimmer
	Loudness	F0 mean/variability	Loudness
	MFCC	Tremor	F0
	Pitch and format measures	Intensity variability	MFCC
		Max phonation time	Formant 1–3 frequency/bandwidth
		Speech/Articulation rate	Harmonic difference
		Pause rate	
	(e.g., 12)	(e.g., 15)	(e.g., 16)

*F0 (Fundamental Frequency)*: the average rate of vocal fold vibrations, determining the perceived pitch of a voice; *Formant 1–3 Frequency/Bandwidth*: the resonance frequencies of the vocal tract (F1, F2, F3) and their bandwidths; *Harmonic Difference*: the amplitude difference between harmonics in a speech signal; *Intensity Variability*: the degree of fluctuation in loudness; *Jitter*: the cycle-to-cycle variation in fundamental frequency; *Loudness*: the perceived intensity of speech; *Max Phonation Time*: the longest duration a person can sustain a vowel sound; *Mel-Frequency Cepstral Coefficients (MFCC):* a representation of the speech spectrum that captures timbral characteristics; *Pause Rate*: the frequency and duration of silent pauses in speech; *Pitch and Formant Measures*: features related to voice pitch and vocal tract resonances; *Shimmer*: the cycle-to-cycle variation in vocal intensity; *Speech/Articulation Rate*: The speed at which speech sounds or syllables are produced; *Tremor*: a rhythmic oscillation in voice frequency or amplitude.

To address these limitations, we leverage a proprietary technology that draws on the foundations of quantitative musicology (Musicology AI). Musicology is the scholarly study of music, encompassing historical, theoretical, and analytical approaches to sound. Applied to voice biomarkers, it involves analyzing speech using music theory concepts such as prosody (rhythm, intonation, stress), acoustic properties (timbre, harmonics, spectral features), and temporal organization (timing patterns, pauses, speech rate). Music theory provides a well-established yet underexplored framework for understanding sound beyond physics, making it a valuable tool for advanced voice analysis. This approach enhances AI models by improving interpretability and biological grounding, for example, speech melodic contours, such as reduced pitch variability, reflect psychomotor retardation in depression, linking to diminished dopaminergic activity (10).

By incorporating musicology into clinical settings, we enhance the accuracy and robustness of voice-based health assessments. However, we recognize that small sample sizes and dataset biases can impact the accuracy, stability, and generalizability of results. To mitigate these challenges, we actively work on the creation of minimally biased large-scale datasets for vocal biomarker research. Additionally, our approach aligns with ensemble learning and weighted feature analysis techniques, incorporating methods such as bootstrap aggregation, boosting, and stacking. These techniques allow us to combine multiple data items, identify the most meaningful feature combinations as hypotheses, and control their influence based on overlapping relationships—enhancing the robustness of our models even in the presence of data biases. By integrating these methods, we further improve the performance, reliability, and fairness of voice-based health assessments, offering a promising path forward in digital diagnostics.

Our most recent technological advancements integrate this knowledge with large language models (LLMs) and fundamental transformer architectures; whereby together with pharmaceutical and quantum hardware partners, we incorporate quantum AI into our core approach. Recent findings from our research (Krautz et al., in prep) further demonstrate the potential of these methods in detecting depressive disorders, showing that musicological features significantly improve the sensitivity and specificity of voice-based depression assessments, reaching AUC = 0.80, also when female and male groups were analysed separately.

Furthermore, one of the fundamental challenges that we identify in the field of mental health diagnostics pertains to the scarcity of high-quality medical voice data. Many existing studies are based on small sample sizes, some are conducted on specific groups of depressed patients, e.g., those with post-traumatic stress disorder (PTSD); which limits the model generalizability. For example, nearly 94% of published studies on depression^2^ involve fewer than 100 participants, and only a small fraction account for complexities such as comorbidities (16%), varying severity levels (13%), or predictive analyses of critical outcomes like death by suicide. This limitation arises partly because obtaining high-quality data from clinical trials is often prohibitively expensive, yet such data is necessary to meet the rigorous requirements for regulatory approval and to provide sufficient ground truth evidence for model performance and efficacy.

To overcome these challenges, we propose a large-scale, data-driven approach that prioritizes both quantity and quality of data. This approach focuses on conducting multicenter studies across diverse linguistic and cultural populations, ensuring that voice biomarkers are validated against real-world clinical diversity. Specifically, in collaboration with one of Europe's largest clinic chains, we launched a multi-site trial spanning two European countries to collect data from a five-digit-amount of patients, encompassing multiple audio recordings per individual alongside comprehensive clinical background information. We currently collect data at several dozens of clinics and aim to ramp up to several hundreds of clinics of our partner. This will create the largest dataset of its kind, significantly improving the robustness of AI models trained for medical voice analysis.

The third pillar of our approach relates to the strong collaboration with world-class clinical partners. That is, we have established contracts entailing more than 400 clinics in Europe enabling us to conduct large-scale, multicenter studies with a diverse patient population. This extensive clinical network ensures access to high-quality data, facilitates standardized study protocols, and enhances the reproducibility and reliability of results. Furthermore, as comparative validation against gold-standard clinical tests is essential for establishing the clinical relevance of voice biomarkers, our studies integrate voice analysis with widely accepted diagnostic tools. In this way we ensure that AI-driven insights are anchored in clinically recognized measures.

The final cornerstone of our approach emphasizes efforts in medical device certification. We are in the process of CE clearance for our voice-based ADHD algorithms. This positions us as front-runner in medical certification. Achieving these certifications, while time and resource consuming, will be a significant milestone in the field of AI-driven medical diagnostics. Regulatory approval necessitates adherence to stringent data integrity, safety, and clinical efficacy standards—challenges that many AI-driven healthcare solutions have struggled to overcome. We therefore pave the way for broader adoption of voice-based diagnostic tools in clinical practice. Before regulatory approval can be achieved, however, it is crucial to establish accuracy and standardization of measurements as a foundation for compliance. Accuracy levels should be aligned with established diagnostic gold standards, such as psychological assessments, and performance metrics must be tailored to specific use cases.

In sum, by combining technological innovation, data scalability, clinical collaboration, and regulatory adherence, we establish a framework for the seamless translation of AI-based voice diagnostics into clinical practice.

## Future research directions

If we move beyond our current research and practice, we recognize that several key research areas must be explored to maximize their clinical impact. Initially, our primary focus is on mental health, with plans to expand into a broader range of diseases and languages over time. While we have already made significant progress in improving the robustness and generalizability of our models, the large-scale data we are currently collecting will elevate these capabilities to an unprecedented level, ensuring even greater clinical reliability. Additionally, integrating voice analysis with other biomarkers, such as physiological data from wearables or facial expressions, presents an opportunity to enhance diagnostic accuracy. This multimodal approach could further refine assessments, particularly in complex conditions like depression and anxiety.

Aforementioned approaches and feature sets aim at establishing evidence for the presence of specific conditions of disease based purely on content agnostic properties of speech. Meanwhile content based analysis are reaching new heights of performance driven by advances in (multilingual) speech to text (STT) conversion and natural language processing (NLP) mainly by the advent of generative pre-training on large copra. Improved STT alone already boosts traditional NLP approaches when analyzing utterance, word and sentence occurrences and relationships. In combination with LLMs, a novel high-level analysis becomes feasible. As an exemplary approach an LLM can be prompted or fine-tuned to assess the contents of speech with respect to the symptoms of a certain psychological condition and to reenact a psychological assessment as either assessor or assesse based on the content presented.

Since content based and content agnostic information has by definition to some degree an orthogonal character, the combination of both has clearly a potential to further improve performance and generalization. An interesting scenario arises when the content of speech points into one direction, say psychological condition negative, while the sub-content or content agnostic properties of the voice hint at condition positive. To some extent the discrepancy between the “what” and the “how” of speech can become a discriminative feature in itself.

An even deeper integration of advanced acoustic perception with pre-trained models with large context awareness could be achieved by feeding aggregated acoustic information directly into the training and inference process. Towards this goal we currently explore the usage of musicological inspired acoustic tokenization to enrich content based tokens.

From an implementation perspective, AI literacy training for healthcare professionals is essential. Medical education programs should incorporate AI-based diagnostic tools, ensuring that clinicians understand their capabilities, limitations, and best practices for integrating them into patient care.

Finally, regulatory frameworks for AI-driven voice biomarkers must evolve to keep pace with technological advancements. Future research should focus on establishing standardized validation protocols, ensuring that AI models meet rigorous clinical efficacy and safety requirements. The ultimate goal is to transition voice biomarkers from promising research tools to fully integrated components of routine medical practice, bridging the gap between innovation and real-world application.

## Conclusion

While AI-driven voice analysis holds promise as a non-invasive biomarker for various health conditions due to its accessibility and ability to facilitate remote monitoring, its adoption in clinical practice, particularly for mental health, remains limited. To fully realize the potential of AI-driven voice analysis in healthcare, a multifaceted approach is required. The integration of proprietary technologies like Musicology AI enhances the depth of voice biomarker analysis, moving beyond conventional methodologies. Addressing the scarcity of high-quality medical voice data through large-scale, multicenter studies ensures that AI models are trained on diverse, representative populations. Strong clinical partnerships provide access to high-quality patient data and facilitate rigorous validation against established clinical scales. Lastly, obtaining medical device certification is vital to adhere to regulatory approval in AI-driven diagnostics. Combining these elements can position voice-based AI as a transformative tool in medical diagnostics.

## Footnotes

^1^In this manuscript, we focus specifically on mental health and depression to make our claims more concrete.

^2^Based on our own review of 67 studies that employed machine learning (ML) and voice analysis.
